# Differences in Metabolite Profiles of Dihydroberberine and Micellar Berberine in Caco-2 Cells and Humans—A Pilot Study

**DOI:** 10.3390/ijms25115625

**Published:** 2024-05-22

**Authors:** Chuck Chang, Yoon Seok Roh, Min Du, Yun Chai Kuo, Yiming Zhang, Mary Hardy, Roland Gahler, Julia Solnier

**Affiliations:** 1 ISURA, Clinical Research, Burnaby, BC V3N 4S9, Canada; cchang@isura.ca (C.C.); kroh@isura.ca (Y.S.R.); mdu@isura.ca (M.D.); rkuo@isura.ca (Y.C.K.); yzhang@isura.ca (Y.Z.); 2 Academy of Integrative and Holistic Medicine, San Diego, CA 92037, USA; mary@maryhardy.com; 3 Factors Group R & D, Burnaby, BC V3N 4S9, Canada

**Keywords:** berberine, dihydroberberine, Caco-2-cells, human study, UHPLC-HRMS, metabolites, micellar, orbitrap

## Abstract

We investigated the pharmacokinetic pathway of berberine and its metabolites in vitro, in Caco-2 cells, and in human participants following the administration of dihydroberberine (DHB) and micellar berberine (LipoMicel^®^, LMB) formulations. A pilot trial involving nine healthy volunteers was conducted over a 24 h period; blood samples were collected and subjected to Ultra High-Performance Liquid Chromatography–High Resolution Mass Spectrometry (UHPLC-HRMS) analyses to quantify the concentrations of berberine and its metabolites. Pharmacokinetic correlations indicated that berberrubine and thalifendine follow distinct metabolic pathways. Additionally, jatrorrhizine sulfate appeared to undergo metabolism differently compared to the other sulfated metabolites. Moreover, berberrubine glucuronide likely has a unique metabolic pathway distinct from other glucuronides. The human trial revealed significantly higher blood concentrations of berberine metabolites in participants of the DHB treatment group compared to the LMB treatment group—except for berberrubine glucuronide, which was only detected in the LMB treatment group. Similarly, results from in vitro investigations showed significant differences in berberine metabolite profiles between DHB and LMB. Dihydroberberine, dihydroxy-berberrubine/thalifendine and jatrorrhizine sulfate were detected in LMB-treated cells, but not in DHB-treated cells; thalifendine and jatrorrhizine-glucuronide were detected in DHB-treated cells only. While DHB treatment provided higher blood concentrations of berberine and most berberine metabolites, both in vitro (Caco-2 cells) and in vivo human studies showed that treatment with LMB resulted in a higher proportion of unmetabolized berberine compared to DHB. These findings suggest potential clinical implications that merit further investigation in future large-scale trials.

## 1. Introduction

Berberine is a natural alkaloid extracted from various plant species, such as European barberry (*Berberis vulgaris*), goldenseal (*Hydrastis canadensis*), goldthread (*Coptis trifolia*), Oregon grape (*Mahonia aquifolium*), phellodendron (*Phellodendron amurense*), and tree turmeric (*Berberis aristata*) [1]. While benzylisoquinoline forms the structural backbone of berberine and many other natural alkaloids, protoberberines are characterized by a tetracyclic ring system with a nitrogen atom sitting on the bridge connecting two sets of bicyclic rings [2]. Berberine is a quaternary ammonium salt from the protoberberines group, and is likely the most widely distributed alkaloid among plant alkaloids [3].

Recently, berberine has garnered significant attention due to its potential therapeutic applications, especially in blood sugar control [4,5], but also in other diseases related to metabolic syndromes [6]. For example, it has shown protective effects against diabetic kidney disease and cardiovascular disease [7,8]. Additionally, it has also shown promise in treating a variety of health conditions such as nonbacterial prostatitis [9], neurological disorders including Alzheimer’s [10,11], and cancer [12,13].

The proposed mechanisms of action for berberine encompass activation of adenosine monophosphate-activated protein kinase (AMPK), regulation of lipid metabolism, enhancement of insulin sensitivity, inhibition of inflammatory pathways, and modulation of gut microbiota [14,15,16,17]. Particularly, the interaction between berberine and gut microbiota has emerged as a focal point of interest, given the growing body of evidence highlighting the significant interplay between the gut microbiome and host physiology [18].

While berberine has demonstrated the ability to improve the metabolic syndrome through modulation of gut microbiota and enhancement of the integrity of the intestinal barrier [16,19,20], the pharmacological activity of berberine is also mediated by its metabolites, which play crucial roles both in direct biological activities and in their interactions with the gut microbiome [21]. Notable among these metabolites are jatrorrhizine, columbamine, demethyleneberberine, berberrubine, and thalifendine, each contributing to the pharmacological effects of berberine through distinct mechanisms [22]. These metabolites arise from various metabolic pathways involving reduction, demethylation, and conjugation reactions, with their glucuronide conjugates adding another layer of complexity to berberine metabolism, thereby influencing its bioavailability and biological activity [23].

Jatrorrhizine, formed through the ring-opening reduction of berberine, exhibits antioxidant, anti-inflammatory, and antimicrobial properties, and has documented effects on gut microbiota and cognitive function [24,25,26]. Columbamine, as a positional isomer of jatrorrhizine, also formed by a ring-opening reduction, shows promise in the treatment of metabolic syndrome and cardiovascular diseases due to its hypolipidemic and hypoglycemic activities [23]. Demethyleneberberine, formed by the ring-opening demethylation of berberine, displays potent anti-inflammatory and neuroprotective effects, suggesting therapeutic potential against inflammatory and neurodegenerative conditions [27,28].

Berberrubine, a major metabolite comprising up to 65.1% of berberine metabolites generated by the liver, is derived from berberine demethylation. It has shown modulatory effects on lipid metabolism and glucose homeostasis, as well as antitumor activity [29,30,31,32,33]. Both berberrubine and its glucuronide can increase glucose metabolism in vitro [34]. Thalifendine, a positional isomer of berberrubine and a primary Phase 1 metabolite of berberine, may also significantly contribute to berberine’s reported therapeutic efficacy [35]. Additionally, glucuronidation of these metabolites represents a major metabolic pathway for berberine to influence its pharmacokinetics and distribution in vivo [1].

Dihydroberberine (DHB) is a hydrogenated derivative of berberine that can be found natively in some plant species, and it could also be derived from berberine by the action of nitroreductase enzymes from gut microbiota [36,37]. When orally ingested, DHB can be converted back to berberine in the gastrointestinal tract by the same enzymes.

Unlike jatrorrhizine, columbamine, demethyleneberberine, berberrubine, and thalifendine, DHB is available as a dietary supplement because it can be chemically synthesized from berberine through a reduction reaction [38]. Additionally, a recent human pharmacokinetic study found that DHB provides higher bioavailability (e.g., ~9 times higher C_max_) than standard berberine in participants [39]. Similarly, a new micellar delivery form of berberine (Berberine LipoMicel) was found to have higher bioavailability than standard formulation in study participants (approx. 10 times higher C_max_). Due to absorption challenges faced by standard berberine treatments, this pilot study aimed to compare these two highly bioavailable formulations, i.e., dihydroberberine (DHB) and micellar berberine (LMB), based on their promising outcomes observed in prior human studies [39,40].

Despite extensive in vivo animal research elucidating the concentrations and distributions of individual berberine metabolites, human clinical data remain limited [41,42]. Thus, this study aims to address this gap by investigating and comparing the metabolite profiles of berberine in both in vitro Caco-2 cells and human participants. Correlating the human pharmacokinetics parameters of various berberine metabolites could help elucidate the metabolic mechanisms involved [43,44]. Positively correlated pharmacokinetics (PKs) could indicate similar metabolic pathways with common metabolizing enzymes and transporters, whereas negatively correlated PKs could indicate competitive metabolism, differential metabolic pathways, or more complex metabolic interactions involving multiple enzymes [45,46].

Furthermore, the current research aims to investigate the discrepancy between berberine metabolite concentrations in human intestinal cells (Caco-2) and human blood, suggesting a potential role for the gut microbiome.

This study is the first to assess the metabolite profiles of two distinct berberine formulations with higher bioavailability—adding to the existing knowledge of berberine metabolism and its potential therapeutic implications.

## 2. Results

### 2.1. Participant Flowchart

**Figure 1:**
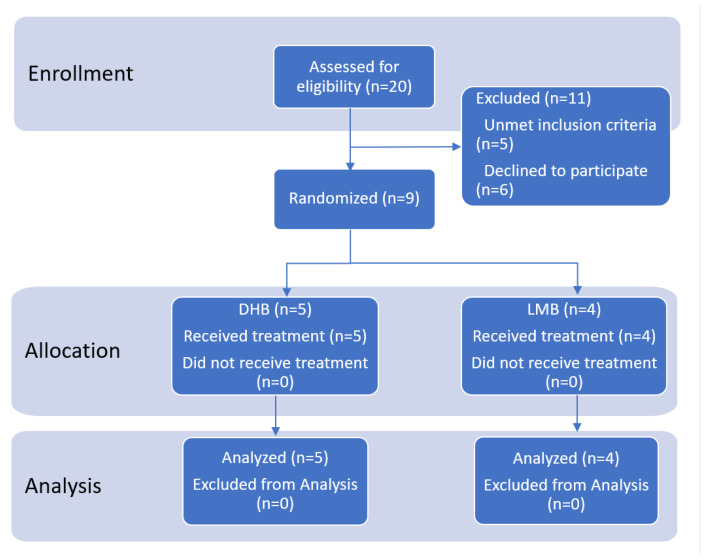
Study participants flow diagram.

Out of twenty participants who were screened for this pilot study, five did not meet the inclusion criteria, and six declined to participate; therefore, nine participants were randomized to parallel treatments in a 2-arm pilot study (Figure 1).

### 2.2. Baseline Characteristics

No significant differences were detected between the two treatment groups at baseline (Table 1), demonstrating that the participants between the two groups were closely matched.

### 2.3. Side Effects

Participants were asked to report the following side effects associated with the treatments on a 5-point scale: bloating, constipation, diarrhea, heartburn, abdominal pain, rash, nausea, dizziness, and vision blurriness.

No adverse events were reported by participants during the study. Both LipoMicel^®^ Berberine and dihydroberberine were well-tolerated at the administered dose.

### 2.4. Pharmacokinetics of Berberine and Metabolites in Human Blood

Berberine metabolites were extracted from full-scan HRMS (High Resolution Mass Spectrometry) data as described in the Materials and Methods section. Table 2 demonstrates the pharmacokinetics (PKs) data over the 24 h study period. DHB achieved higher blood concentrations of berberine and berberine metabolites except for berberrubine glucuronide. Notably, berberrubine glucuronide was not detected in the DHB group, even though a higher concentration of its aglycone was found in the DHB group compared to the LMB group (C_max_ 1.3 ± 0.4 vs. 0.09 ± 0.03 ng/mL). As expected, dihydroberberine concentrations in blood following DHB administration were several times higher following administration of DHB compared to LMB (AUC_0–24_ 1.2 ± 0.5 vs. 0.05 ± 0.03 ng·h/mL; Table 2). Similarly, berberine concentrations were approx. 1.6-fold higher following the oral administration of DHB compared to LMB (AUC_0–24_ 41.1 ± 7.0 vs. 26.0 ± 14.2 ng·h/mL; Table 2); however, no significance was found due to the small sample size. The T_max_ values of berberine for DHB and LMB were different (~1 h vs. 11 h), which may indicate a slower metabolism and different absorption mechanism of micellar LMB.

Post hoc analyses revealed significant differences between the AUC_0–24_ values of berberrubine, demethyleneberberine, and berberrubine glucuronide between the two treatments.

#### Pharmacokinetic Correlation of Metabolites

To determine how the metabolites relate to one another in their pharmacokinetic (PK) parameters, a correlation matrix was created based on their AUC_0–24_, C_max_, and T_max_ (Table 3). Berberine PK had a strong positive correlation with thalifendine, weak positive correlation with DB, DB-SO4, and negative correlation with dihydroxyberberine, dihydroxy-berberrubine/thalifendine, columbamine glucuronide, jatrorrhizine-glucuronide, thalifendine glucuronide, and DB glucuronide. Dihydroberberine PK had a strong positive correlation with berberrubine, columbamine, columbamine-glucuronide, thalifendine-glucuronide, and demethyleneberberine glucuronide.

Berberrubine sulfate and thalifendine sulfate were strongly and significantly correlated to demethyleneberberine sulfate, but was only weakly correlated to jatrorrhizine sulfate. Columbamine glucuronide correlated strongly and significantly with jatrorrhizine glucuronide, thalifendine glucuronide, and demethyleneberberine glucuronide, but only weakly with berberrubine glucuronide. Jatrorrhizine sulfate correlated strongly and significantly with dihydroxyberberine, dihydroxy-berberrubine/thalifendine, columbamine-glucuronide, demethyleneberberine glucuronide. Berberrubine/thalifendine sulfate correlated strongly and significantly with berberrubine and demethyleneberberine sulfate.

### 2.5. Metabolites from Caco-2 Cell Cultures

**Figure 2:**
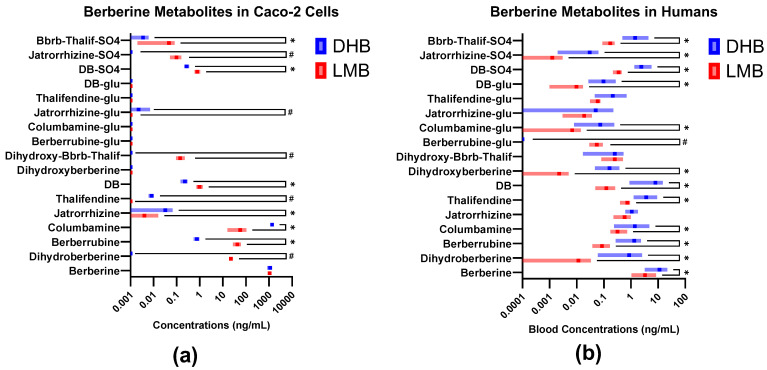
Comparison of ranges (min to max) of concentrations of berberine metabolites between dihydroberberine (DHB, blue) and LipoMicel^®^ Berberine (LMB, red). (**a**) Berberine metabolites in Caco-2 cells after 2 h of incubation. (**b**) Berberine metabolites in human whole blood. C_max_ data presented. Dark bars indicate the mean concentrations observed while light-colored bars indicate the range of concentrations observed. * Indicates q ≤ 0.05 with a false discovery rate of 5% using the paired *t*-test (Caco-2 data) or unpaired Mann–Whitney test (human data). # indicates the metabolite was not detected in one treatment. Glu = glucuronide. See Table S1 for other compound abbreviations.

Glucuronides of berberine metabolites were not detected in the basal culture media of either treatment on Caco-2 cells (Figure 2a).

#### Comparison with Human Blood

Figure 2 and Table S2 highlight the differences between the concentrations of berberine and its metabolites detected in Caco-2-cells vs. human blood.

**Figure 3:**
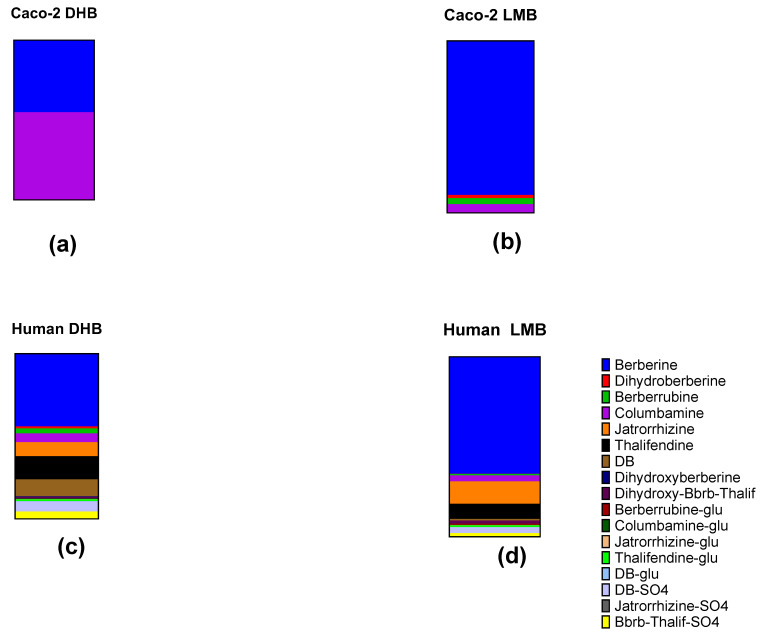
Proportional representation of concentrations of berberine metabolites detected (**a**) in Caco-2-cell culture media treated with DHB, (**b**) in Caco-2-cell culture media treated with LMB, (**c**) in human participants treated with DHB, and (**d**) in human participants treated with LMB. Glu = glucuronide. See Table S1 for other compound abbreviations.

Except for dihydroxyberberrubine and berberrubine glucuronide, blood from participants in the DHB treatment group provided concentrations that are several folds higher than that from the LMB treatment group (Table S2). However, when viewed proportionally, a greater fraction of total metabolites in the blood of participants in the LMB treatment group remained as berberine compared to the DHB treatment group (Figure 3c,d). This is consistent with the higher proportion of berberine observed in LMB-treated Caco-2 cells compared to DHB-treated cells.

## 3. Discussion

While prior investigations have suggested comparable high bioavailability between the two berberine formulations, DHB and LMB, in contrast to standard berberine [39,40], the present study is the first to unveil notable disparities in their metabolite profiles in both Caco-2 cell cultures and human subjects. This integration of data from the human trial and the in vitro cell culture experiments underscores a pivotal strength of our present investigation.

The correlation matrix analysis of pharmacokinetic parameters pertaining to various metabolites has yielded intriguing and unexpected outcomes. Notably, despite berberrubine and thalifendine being positional isomers, and both arising from berberine demethylation [23], our observations indicated a strong positive correlation between the pharmacokinetics of berberine and thalifendine, while berberrubine displayed no such correlation (Table 3). This discrepancy suggests potential disparate metabolic pathways for these two metabolites owing to their structural differences. Notably, the robust correlation observed between the pharmacokinetics of berberrubine sulfate/thalifendine sulfate and demethyleneberberine sulfate, coupled with their weak correlation with jatrorrhizine sulfate, hinted at potential divergence in the metabolic pathway leading to the production of jatrorrhizine sulfate despite its structural similarity to the other two sulfate conjugates (Table 3). Among the glucuronide conjugates, weak correlations were observed for the pharmacokinetics of berberrubine glucuronide with columbamine glucuronide, jatrorrhizine glucuronide, thalifendine glucuronide, and demethyleneberberine glucuronide, in contrast to the strong inter-correlation among the latter four (Table 3), which suggested the possibility of a unique metabolic pathway for berberrubine glucuronide.

In human blood, significant differences in PK parameters such as AUC_0–24_ (*p* = 0.002), C_max_ (*p* = 0.032), and T_max_ (*p* < 0.001) of certain berberine metabolites were observed (Table 2); post hoc analysis revealed that the AUC_0–24_ values of berberrubine, demethyleneberberine, and berberrubine glucuronide differed significantly between the two treatments (Table 2). Previous studies have highlighted the potential of both berberrubine and berberrubine glucuronide in enhancing glucose uptake and consumption, with berberrubine also showing promise in restoring gut microbiota [34,47]. Notably, while DHB treatment resulted in higher berberrubine concentrations in human blood, its concentrations in the basal media of Caco-2 cultures were lower compared to LMB (Figure 3 and Table S2). A similar trend was observed with demethyleneberberine—a derivative that has been associated with alleviating symptoms of inflammatory bowel disease in previous studies [27,48].

The discrepancies in metabolite concentrations between DHB and LMB could have implications for future clinical studies investigating their pharmacological effects on glucose metabolism and on intestinal conditions involving the gut microbiota. For example, in the case of berberrubine, since the DHB formulation resulted in similar concentrations in Caco-2 culture media and in human blood (0.75 ± 0.10, 1.3 ± 0.4 ng/mL, respectively; Table 2), but the same comparison for the LMB formulation showed a 500-fold difference (43 ± 7, 0.085 ± 0.03 ng/mL, respectively for Caco-2 and blood; Table S2), would LMB treatment result in more berberrubine being available for use by the gut microbiota, as opposed to allowing more of this metabolite to pass into circulation? Would LMB treatment also result in greater restoration of gut microbiota? Future studies could be designed to investigate such questions.

The positional isomers jatrorrhizine and columbamine and their glucuronides showed similar patterns between DHB and LMB under Caco-2 culture and in human blood, which can be expected due to their structural similarities. (Figure 3). The same pattern was observed for another pair of positional isomers, berberrubine and thalifendine, in human blood but not in Caco-2 culture; their respective glucuronides also showed different patterns in human blood. Despite similar relative concentrations of berberrubine and thalifendine, berberrubine glucuronide was undetected in human blood following the DHB treatment, whereas it was detected in the blood of LMB-treated participants (Figure 3 and Table S2). Again, this might point to unique biological pathways for the production and excretion of berberrubine glucuronide.

Interestingly, we observed a substantial generation of dihydroberberine as a metabolite via human Caco-2 cells that were treated with LMB (Table S2). While a previous study had demonstrated that dihydroberberine was produced by incubating berberine with murine or human intestinal microbes [37], this process can apparently occur independently of microbial nitroreductases. This finding may help explain the detection of dihydroberberine in the blood and feces after oral administration in view of berberine’s inherent antimicrobial properties [49,50].

The metabolite profiles of DHB and LMB in Caco-2 cells markedly differ from those in human whole blood (Figure 2 and Figure 3). These differences could be a result of the in vitro models not fully replicating the complex mechanisms in the human intestines. For example, specific compounds may be hindered by the mucosal barrier, metabolized or effluxed by the gut wall or microbiota, form complexes with luminal contents, or rapidly transit through the gastrointestinal tract, thereby reducing their absorption. Additionally, metabolic processes and enzymatic activities taking place in the liver such as CYP enzyme activity could further account for the final metabolite profile of berberine or dihydroberberine detected in circulating blood [30,51]. In most cases, metabolites that were low in concentration in the blood but were high in concentration when incubated with Caco-2 cells were likely poorly absorbed through the intestinal membrane. Another possibility for differing metabolite profiles is the differential dissolution potential of LMB and DHB in blood plasma. However, since DHB is freely soluble in water, while LMB’s solubility is limited in water (2.34 mg/mL) and intestinal conditions (4.04 mg/mL) [40], DHB would theoretically provide greater systemic exposure, leading to a more reliable therapeutic response. If one assumes the therapeutic efficacy of berberine to be derived from direct absorption, the higher blood concentrations of most metabolites in DHB-treated participants would support this theory (Table 2 and Figure 2b).

Previous research has suggested that the gut microbiome plays a significant role in the metabolism and potentiation of biological effects of berberine in humans [17,21]. One proposed mechanism for the blood glucose lowering effects of berberine is through its modulation of the gut microbiota [52], as demonstrated in animal models of diabetes [53,54]. Additionally, oral dosing of berberine has been shown to promote the growth of *Akkermansia* by stimulating mucin production in the colon [55], and *Akkermansia* enrichment has been associated with the retardation of diabetic disease progression [56,57]. Since structural analogs of berberine have different effects on the gut microbiome [58] and berberine undergoes extensive Phase 1 metabolism in the intestinal mucosa [59], it can be concluded that interactions of berberine and its metabolites with gut microbiome is an important part of berberine’s mechanism of action [17,19,60]. In fact, experiments on human gut microbiomes cultured in vitro showed that while berberine and dihydroberberine strongly enriched *Akkermansia* and *Bacteroides* spp., jatrorrhizine, columbamine, and demethyleneberberine had a lowering or negative effect on the growth of these bacteria [58].

The current study showed that under LMB treatment, a higher proportion of berberine remained unmetabolized compared to DHB treatment, both in vitro and in vivo (Figure 3). Thus, LMB treatment could lead to greater enrichment of *Akkermansia* and *Baceteroides* spp. compared to DHB. While the effect of the two formulations on human gut microbiota was beyond the scope of the current study, future research could explore this further by determining the changes in the compositions of the gut microbiome before and after berberine treatment, possibly also through the co-administration of a predefined mixture of probiotics.

Certain clinical aspects of berberine depend on the delivery of unmetabolized berberine to the digestive tract instead of the circulatory system. For instance, a randomized trial found that berberine given at 0.3 g twice per day reduced the risk of colorectal carcinoma recurrence [61], possibly due to its strong affinity with formate tetrahydrofolate ligase, inhibiting the growth of serval colorectal cancer-driving bacteria as well as human tumor cells [62]. Given that this binding affinity is dependent on berberine’s molecular structure, the higher proportion of unmetabolized berberine retained in LMB treatment could be advantageous.

Limitations of this pilot study include the small number of human participants, as well as a short observation period of 24 h. For instance, there was a considerable difference between the T_max_ values of DHB vs. LMB treatment, likely due to the higher lipophilicity of LMB compared to DHB [63].

The findings from the current study may provide guidance for the design of future large-scale clinical trials aiming to correlate metabolites of berberine with clinical outcomes. Further research is warranted to elucidate the clinical implications of the observed differences in metabolite profiles between DHB and LMB treatments, particularly in the context of gut microbiota modulation and therapeutic efficacy in metabolic and intestinal conditions.

## 4. Materials and Methods

### 4.1. Study Treatments

LipoMicel^®^ Berberine was provided by Natural Factors (Coquitlam, BC, Canada), and dihydroberberine, sold as Gains Candy GlucoVantange (Alpha Lion, New York, NY, USA), was purchased online from www.nutritionfaktory.com (accessed on 13 September 2023). Each LipoMicel Berberine capsule contains 250 mg of berberine (hydrochloride) from *Berberis vulgaris* in a soft-gelatin capsule. Each GlucoVantage capsule contains 100 mg of dihydroberberine with 5 mg of black pepper extract in a 2-piece hard gelatin capsule. The compositions of the study treatments were verified by UHPLC analyses (Figure S2). Different dosages of the two treatments were used because dihydroberberine provides several fold higher blood concentrations of berberine compared to standard berberine [39].

### 4.2. Study Participants

Participants were included in the study if they met the following criteria: 21 years old or older, non-smokers, and not taking any prescription medication. Participants were excluded if they had any serious acute or chronic diseases such as liver, kidney, or gastrointestinal diseases, or if they were allergic to berberine. Furthermore, women who were pregnant, planning to become pregnant, or breast-feeding were excluded.

Study participants were randomized using the RAND function of Microsoft Excel 2016. The randomization file was kept by the study coordinator and unblinded after data analysis.

### 4.3. Study Design

A double-blind, randomized pilot study was conducted at ISURA’s research facilities in Burnaby, British Columbia, Canada. This study was approved by the Canadian SHIELD Ethics Review Board with Office for Human Research Protections (OHRP) Registration IORG0003491 under REB Tracking Number 2021-08-004. The study is registered on ClinicalTrial.gov with identifier NCT06202157. All participants signed an informed consent prior to the study.

From 48 h before the start of each treatment, participants were asked to refrain from consuming any supplements for food containing berberine or dihydroberberine. There were no other restrictions on dietary supplement intake.

**Figure 4:**
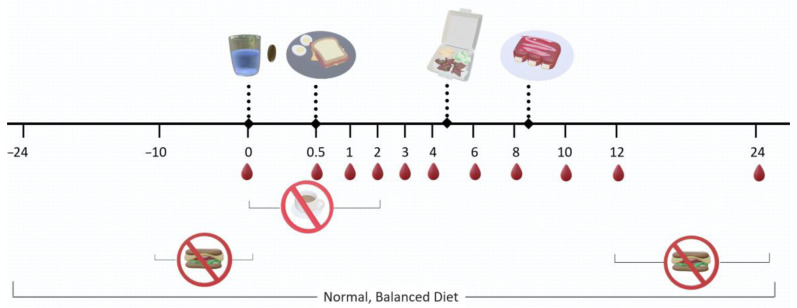
Treatment progress diagram. Participants were asked to fast for at least 8 h prior to the first and the last blood sample. No tea or coffee was consumed during the first 2 h of the study. A standardized breakfast, lunch, and dinner were provided (after 0.5, 4 and 8 h post-intervention, respectively). Eleven blood samples were taken over a period of 24 h.

On the day of the study, participants arrived after an overnight fast, and then were given a small cup labeled with their name containing one of two randomly preassigned interventions. A baseline blood sample (t = 0) was collected from each participant prior to the intervention, and subsequent blood samples were collected at 0.5, 1, 2, 3, 4, 6, 8, 10, 12, and 24 h post-intervention. Participants were given a standardized breakfast consisting of a ham and cheese sandwich with a hardboiled egg, as well as a standardized lunch and dinner after 4 h and 8 h, respectively (Figure 4).

### 4.4. Blood Collection and Sample Preparation

Capillary blood from the participants was collected using sterile, single-use lancets (Trividia Health, Fort Lauderdale, FL, USA) along with a 50-µL collection tube containing potassium EDTA as anticoagulant (Sarstedt, Nümbrecht, NRW, Germany). Blood samples were kept frozen at −20°C until analysis. On each day of sample analyses, blood samples were thawed at room temperature, and benzanilide (Sigma-Aldrich, St. Louis, MO, USA) was added to 50 µL of whole blood as an internal standard. Next, the samples were extracted with 250 µL of High-Performance Liquid Chromatography (HPLC)-grade methanol (Thermo Fisher Scientific Inc., Waltham, MA, USA) by submerging extraction tubes in a sonicating water bath for 30 min. Extracted samples were spun in a centrifuge at 10,000× *g* for 5 min, and then the supernatant was pipetted into a 96-well microplate for berberine and metabolites analyses.

### 4.5. Cell Cultures

Caco-2 cells (Cedarlane Laboratories, Toronto, ON, Canada) were cultured in a T-25 flask (Thermo Fisher Scientific Inc., Waltham, MA, USA) using a HERACELL VIOS 160i CO_2_ incubator (Thermo Fisher Scientific Inc., Waltham, MA, USA) set to 37 °C and 5.0% CO_2_. The composition of the cell culture media was as follows: Dulbecco’s modified Eagle’s medium (DMEM) (Sigma-Aldrich, St. Louis, MO, USA), 10% heat-inactivated fetal bovine serum (FBS) (Thermo Fisher Scientific Inc., Waltham, MA, USA), penicillin (100 units/mL), and streptomycin (100 units/mL) (Sigma-Aldrich, St. Louis, MO, USA). Cells were resuspended with 5% trypsin (Thermo Fisher Scientific Inc., Waltham, MA, USA), and seeded on a 24-well format polycarbonate semipermeable membrane insert with a diameter of 6.5 mm and a pore size of 0.4 μm (VWR International, Toronto, ON, Canada). The seeding density was 1 × 10^4^ cells/cm^2^. Seeded cells were incubated with the culture media for 21 days so that they can homogeneously differentiate and form monolayers; then, the cells were processed for the metabolites assay.

On the day of treatment, Caco-2 cells were washed twice with Hanks’ balanced salt solution (HBSS) (Sigma-Aldrich, St. Louis, MO, USA), and then allowed to equilibrate for 30 min in the incubator at 37 °C. Next, LMB, DHB, or cell culture media serving as negative control was added as donor solutions to the apical side of the monolayer, and 500 µL of HBSS solution to the basal side. Four hours after the treatment, the basal solution was collected for metabolites analysis. Extraction of metabolites from the basal solutions followed the same procedure as the blood samples, starting with the addition of benzanilide. All treatments were performed in triplicates. No detectable levels of berberine or berberine metabolites were found in the negative controls.

### 4.6. Analysis of Berberine and Metabolites

Blood and cell culture samples were analyzed for berberine and its metabolites with a validated method utilizing Ultra High-Performance Liquid Chromatography coupled with High Resolution Mass Spectrometry (UHPLC-HRMS), as previously described [40,64,65,66].

Extracted samples were injected at 20 µL volumes into a Vanquish UHPLC (Ultra High Pressure Liquid Chromatography) system (Thermo Fisher Scientific Inc., Waltham, MA, USA) from microplates kept at 10.0 °C and separated on Acme Xceed C18 columns (100 × 2.1 mm, 1.9 µm, Phase Analytical Technology, State College, PA, USA) that were temperature-controlled in a 40.0 °C column oven. A binary mobile phase consisted of 0.5% formic acid in water (Mobile Phase A) and methanol (Mobile Phase B) was used with a linear gradient from 10–100% Mobile Phase B over 4.00 min to elute the analytes of interest. All reagents used were Liquid Chromatography Mass Spectrometry (LCMS) grade and obtained from Fisher Chemicals, ON, Canada.

The HRMS (Q Exactive^TM^ Orbitrap^TM^, Thermo Fisher Scientific Inc., Waltham, MA, USA) was set to scan from 150 to 1000 *m*/*z* at 70,000 mass resolution in Full MS–SIM mode using a heated electrospray ion source (HESI) operated in positive mode. Typical mass errors were less than 1 ppm over a one-week period.

Berberine and its metabolites were determined using berberine HCl (Sigma-Aldrich, St. Louis, MO, USA) for calibration against benzanilide as an internal standard. HRMS data were extracted at 5 ppm mass tolerance windows according to Table S1. See Figure S1 for sample chromatograms.

### 4.7. Data Analysis and Statistical Methods

Descriptive statistics were applied using GraphPad Prism 10.1 software (Boston, MA, USA). AUCs were calculated using the trapezoid method. Data were considered significant at *p* ≤ 0.05. Unless otherwise indicated, results are expressed as mean ± standard error of measurement (SEM).

Participants’ baseline characteristics were evaluated with 2-way ANOVA using Šídák’s multiple comparisons test. Caco-2 metabolite comparisons were made using multiple paired *t*-test statistics with a False Discovery Rate (FDR) approach with the two-stage step-up method of Benjamini, Krieger and Yekutieli, and a desired FDR (*q*) of 5%. The correlation matrix of human pharmacokinetics (PK) data (AUC_0–24_, C_max_, and T_max_) was created from group covariance matrices after performing two-factors MANOVA on data normalized through log(x + 1) transformation. [Real Statistics Resource Pack Software Release 7.6. Copyright 2013–2021. Charles Zaiontz. www.real-statistics.com (accessed on 11 May 2022)]. The resource pack was an add-on software package for Microsoft Excel.

## 5. Conclusions

This study found evidence suggesting that berberrubine, jatrorrhizine sulfate, and berberrubine glucuronide may follow distinctive metabolic pathways compared to structurally similar metabolites of berberine, which could have implications for future research on these compounds. Furthermore, our findings provided valuable insights into the distinct metabolite profiles of two high-bioavailability formulations, dihydroberberine (DHB) and LipoMicel^®^ Berberine (LMB), both in vitro and in human participants. In addition to displaying distinct metabolite profiles, LipoMicel treatment demonstrated a higher retention of unmetabolized berberine following Phase 1 and Phase 2 metabolism in both human participants and Caco-2 cell cultures compared to dihydroberberine. This observation may hold significant clinical implications, particularly in terms of blood glucose regulation in humans. Consequently, further investigations into the pharmacological actions of specific metabolites will be important for enhancing the understanding of berberine’s therapeutic efficacy.

## Figures and Tables

**Table 1:** Baseline characteristics.

Parameter	DHB	LMB	*p*-Value
*n*	5	4	
Males|Females	2|3	3|1	
Age (years)	38.0 ± 5.4	36.0 ± 4.2	0.9995
Weight (kg)	67.0 ± 3.9	64.0 ± 2.9	0.9890
BMI (kg/m^2^)	23.7 ± 0.4	21.7 ± 0.9	0.9207
Fasting Blood Glucose (mmol/L)	5.39 ± 0.22	5.64 ± 0.34	0.9995
Total Cholesterol (mmol/L)	4.49 ± 0.58	5.17 ± 0.41	>0.9999
Triglyceride (mmol/L)	0.87 ± 0.15	1.82 ± 0.72	>0.9999
High Density Lipoprotein (mmol/L)	1.60 ± 0.22	1.50 ± 0.14	>0.9999
Low Density Lipoprotein (mmol/L)	2.50 ± 0.42	2.84 ± 0.32	>0.9999

Data is presented as means ± SEM. No significant differences were observed with 2-way ANOVA using Šídák’s multiple comparisons test.

**Table 2:** Pharmacokinetics parameters of berberine metabolites in humans.

Metabolites	AUC_0–24_ (ng·h/mL)		C_max_ (ng/mL)		T_max_ (h)	
	DHB	LMB	DHB	LMB	DHB	LMB
Berberine	41.1 ± 7.0	26.0 ± 14.2	11.2 ± 3.0	3.3 ± 1.7	1.2 ± 0.5	10.5 ± 5.0
Dihydroberberine	1.2 ± 0.5	0.05 ± 0.03	0.85 ± 0.45	0.01 ± 0.01	1.6 ± 0.4	2.5 ± 1.3
Berberrubine	2.9 ± 1.0 *	0.33 ± 0.11 *	1.3 ± 0.4	0.09 ± 0.03	1.6 ± 0.2	5.3 ± 2.2
Columbamine	4.8 ± 2.8	1.3 ± 0.5	1.4 ± 0.8	0.32 ± 0.13	2.4 ± 0.9	3.5 ± 0.9
Jatrorrhizine	7.9 ± 0.8	5.0 ± 1.0	1.1 ± 0.3	0.58 ± 0.16	10.4 ± 3.7	7.8 ± 2.0
Thalifendine	13.2 ± 4.4	3.4 ± 0.8	3.6 ± 1.4	0.73 ± 0.12	2.4 ± 0.9	5.5 ± 1.7
DB	9.6 ± 2.1 *	0.33 ± 0.15 *	7.9 ± 2.7 *	0.12 ± 0.05 *	1.6 ± 0.4	2.8 ± 1.1
Dihydroxyberberine	0.34 ± 0.11	0.003 ± 0.002	0.16 ± 0.06	0.002 ± 0.001	2.4 ± 0.9	2.3 ± 1.9
Dihydroxy-Bbrb/Thalif	0.95 ± 0.46	0.75 ± 0.35	0.25 ± 0.10	0.25 ± 0.10	6.3 ± 4.5	4.3 ± 2.3
Berberrubine-glu	ND *	0.27 ± 0.04 *	ND *	0.06 ± 0.01 *	ND *	2 ± 0 *
Columbamine-glu	0.27 ± 0.15	0.02 ± 0.01	0.17 ± 0.14	0.007 ± 0.003	2.0 ± 0.4	3.3 ± 1.3
Jatrorrhizine-glu	0.18 ± 0.13	0.06 ± 0.02	0.14 ± 0.14	0.02 ± 0.01	1.4 ± 0.7	1.5 ± 0.3
Thalifendine-glu	1.1 ± 0.4	0.40 ± 0.09	0.54 ± 0.45	0.06 ± 0.01	3.4 ± 0.7	4.8 ± 0.8
DB-glu	0.21 ± 0.11	0.03 ± 0.01	0.17 ± 0.12	0.010 ± 0.004	1.6 ± 0.4	2 ± 0
DB-SO4	5.7 ± 1.9	1.30 ± 0.05	3.9 ± 2.3 *	0.35 ± 0.05 *	1.6 ± 0.4	2 ± 0
Jatrorrhizine-SO4	0.066 ± 0.035	0.002 ± 0.001	0.045 ± 0.025	0.001 ± 0.001	1.8 ± 0.5	1.0 ± 0.6
Bbrb/Thalif-SO4	3.8 ± 2.0	0.70 ± 0.11	2.9 ± 2.2	0.17 ± 0.04	1.6 ± 0.4	2.3 ± 0.3

“ND” indicates the metabolite was not detected. “glu” = glucuronide. See Table S1 for other compound abbreviations. *p*-values were calculated using Two-Factor MANOVA. Treatment factor: *p* = 3.98 × 10^−5^, Wilks’ lambda = 0.0031. * denotes significant differences between groups based on Bonferroni confidence interval at alpha = 0.05.

**Table 3:** Correlation matrix of the pharmacokinetic parameters of berberine metabolites.

Metabolites	1	2	3	4	5	6	7	8	9	10	11	12	13	14	15	16	17
1. Berberine																	
2. Dihydroberberine	0.08																
3. Berberrubine	0.27	0.74 *															
4. Columbamine	0.35	0.75 *	0.62 *														
5. Jatrorrhizine	0.28	0.58	0.55	0.61 *													
6. Thalifendine	0.73 *	0.48	0.54	0.76 **	0.54												
7. DB	0.53	0.54	0.61 *	0.51	0.23	0.67 *											
8. Dihydroxyberberine	−0.12	0.53	0.39	0.37	0.57	0.25	0.09										
9. Dihydroxy-Bbrb/Thalif	−0.10	0.42	0.23	0.44	0.60 *	0.30	−0.11	0.73 *									
10. Berberrubine-glu	0.01	0.25	0.46	0.28	0.24	0.14	−0.12	0.17	0.28								
11. Columbamine-glu	−0.22	0.77 **	0.67 *	0.46	0.58 *	0.16	0.06	0.75 **	0.60 *	0.48							
12. Jatrorrhizine-glu	−0.28	0.45	0.60 *	0.28	0.28	0.03	0.00	0.51	0.19	0.48	0.76 **						
13. Thalifendine-glu	−0.19	0.62 *	0.75 **	0.57	0.65 *	0.26	0.16	0.63 *	0.55	0.53	0.81 **	0.71 *					
14. DB-glu	−0.34	0.62 *	0.65 *	0.47	0.58	0.11	0.06	0.69 *	0.62 *	0.56	0.86 **	0.72 *	0.94 ***				
15. DB-SO4	0.51	0.38	0.63 *	0.51	0.42	0.65 *	0.65 *	0.11	0.05	−0.04	0.19	0.15	0.37	0.21			
16. Jatrorrhizine-SO4	−0.41	0.51	0.28	0.36	0.52	0.08	−0.11	0.78 **	0.76 **	0.18	0.77 **	0.55	0.64 *	0.79 **	0.05		
17. Bbrb/Thalif-SO4	0.30	0.49	0.76 **	0.54	0.48	0.51	0.52	0.26	0.15	0.15	0.45	0.40	0.63 *	0.49	0.92 ***	0.24	

Numbers in the header row correspond to metabolites in the first column. Glu = glucuronides. Please see Table S1 for other compound abbreviations. Correlations of AUC_0–24_, C_max_, and T_max_ were based on data from all participants (*n* = 9). Significance level (2-tailed *t*-tests): * *p* < 0.05; ** *p* < 0.01; *** *p* < 0.001.

## Data Availability

The data presented in this study are available on request from the corresponding author. The data are not publicly available due to ethical restrictions.

## Supplementary Materials

The following supporting information can be downloaded at: https://www.mdpi.com/article/10.3390/ijms25115625/s1.
