# Supplementary material for: Differences in Metabolite Profiles of Dihydroberberine and Micellar Berberine in Caco-2 Cells and Humans—A Pilot Study
Source: Int J Mol Sci. 2024 May 22;25(11):5625. doi: 10.3390/ijms25115625 (PMC11171481; doi:10.3390/ijms25115625)
Supplement: Supplementary file 1 [file ijms-25-05625-s001.zip › ijms-2990338-supplementary.pdf]

Table S1. HRMS data extraction parameters for berberine and its metabolites

Compound Name	Compound Formula	Adduct	m/z	Retention Time
Benzanilide	C13H11NO	M+H	198.0913	3.78
Berberine	C20H18NO4	M+H	336.1230	2.38
Jatrorrhizine	C20H20NO4	M	338.1387	2.02
Columbamine	C20H20NO5	M	338.1387	2.29
Dihydroberberine	C20H19NO4	M+H	338.1387	2.73
Demethyleneberberine (DB)	C19H18NO4	M+H	324.1230	2.06
Demethyleneberberine Glucuronide (DB-glu)	C25H26NO10	M+H	500.1551	1.93
Jatrorrhizine Glucuronide	C26H28NO10	M+H	514.1708	2.03
Columbamine Glucuronide	C26H28NO10	M+H	514.1708	2.10
Berberrubine	C19H16NO4	M+H	322.1074	2.86
Thalifendine	C19H16NO4	M+H	322.1074	2.25
Berberrubine Glucuronide	C25H24NO10	M+H	498.1395	2.19
Thalifendine Glucuronide	C25H24NO10	M+H	498.1395	1.99
Berberrubine or Thalifendine Sulfate (Bbrb/Thalif SO4)	C19H16NO7S	M+H	402.0642	2.85
Demethyleneberberine Sulfate (DB SO4)	C19H16NO7S	M+H	404.0798	2.75
Jatrorrhizine 3-O-Sulfate (Jatrorrhizine SO4)	C20H19NO7S	M+H	418.0955	2.75
Dihydroxy- Berberrubine or Thalifendine (dihydroxy-Bbrb/Thalif)	C19H16NO6	M+H	354.0972	2.76
Dihydroxyberberine	C20H18NO6	M	368.1129	2.07

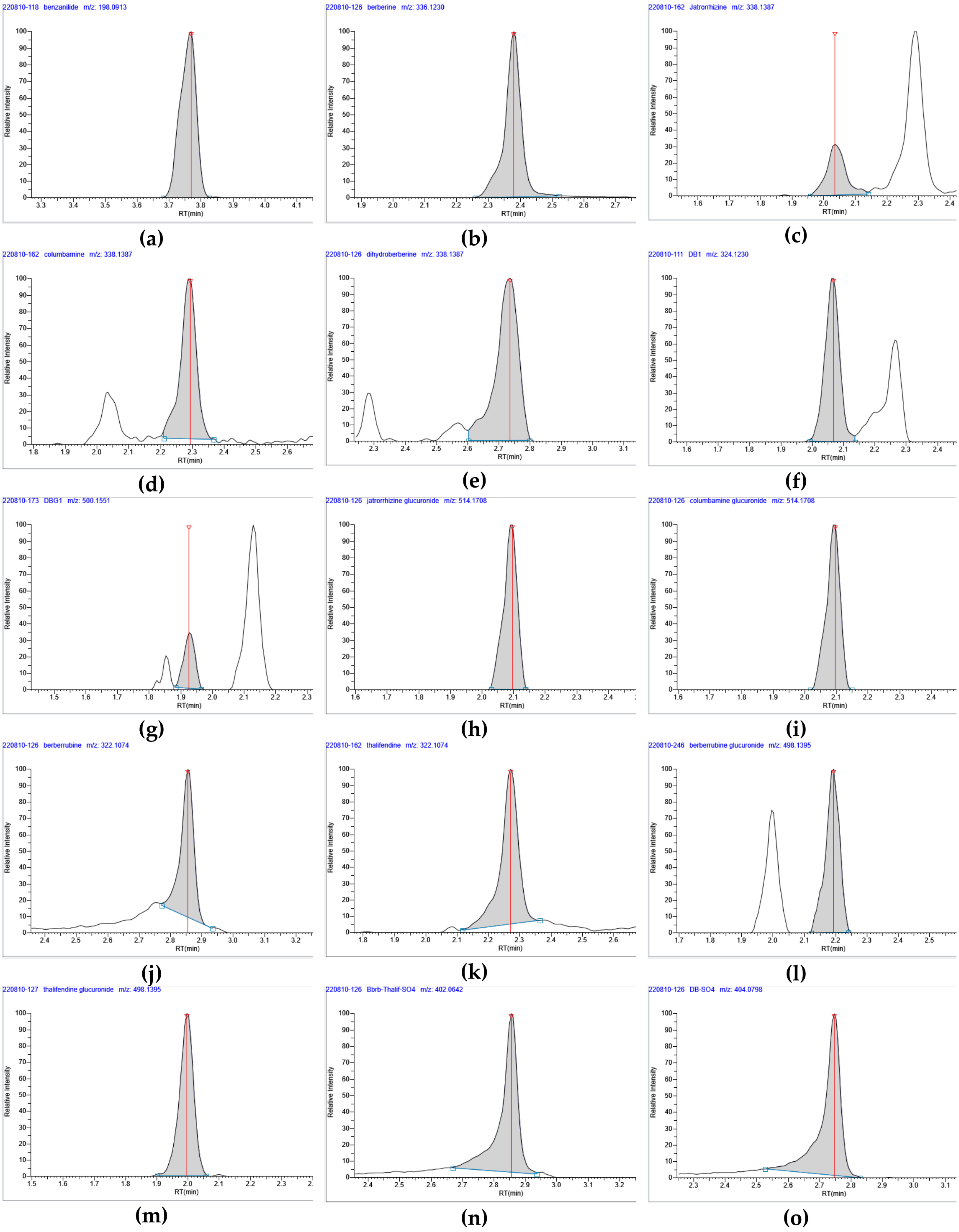
220810-118 benzanilide m/z: 198.0913
220810-126 berberine m/z: 336.1230
220810-162 Jatrorrhizine m/z: 338.1387
(a)
(b)
(c)
220810-162 columbamine m/z: 338.1387
220810-126 dihydroberberine m/z: 338.1387
220810-111 DB1 m/z: 324.1230
(d)
(e)
(f)
220810-173 DBG1 m/z: 500.1551
220810-126 jatrorrhizine glucuronide m/z: 514.1708
220810-126 columbamine glucuronide m/z: 514.1708
(g)
(h)
(i)
220810-126 berberrubine m/z: 322.1074
220810-162 thalifendine m/z: 322.1074
220810-246 berberrubine glucuronide m/z: 498.1395
(j)
(k)
(l)
220810-127 thalifendine glucuronide m/z: 498.1395
220810-126 Bbrb-Thalif-SO4 m/z: 402.0642
220810-126 DB-SO4 m/z: 404.0798
(m)
(n)
(o)
Relative Intensity
RT(min)

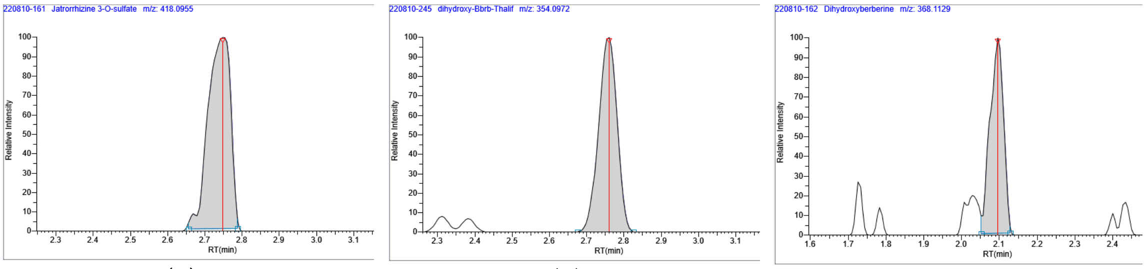

(p) **(q)** **(r)**

Figure S1: LC-HRMS chromatograms of berberine and its metabolites. (a) benzanilide (internal standard), (b) berberine, (c) jatrorrhizine, (d) columbamine, (e) dihydroberberine, (f) demethyleneberberine, (g) demethyleneberberine glucuronide, (h) jatrrohizine glucuronide, (i) columbamine glucuronide, (j) berberrubine, (k) thalifendine, (l) berberrubine glucuronide, (m) thalifendine glucuronide, (n) berberrubine or thalifendine sulfate, (o) demethyleneberberine sulfate, (p) jatrorrhizine 3-o-sulfate, (q) dihydroxy-berberrubine or thalifendine, and (r) dihydroxyberberine

Table S2. Summary of observed differences in DHB and LMB treatments in Caco-2 metabolites and Human blood metabolites.

Compound Name	Chemical Structure	Concentrations in Caco-2 cell (ng/mL)		Concentrations in Human Blood (ng/mL)	
		DHB	LMB	DHB	LMB
Dihydroberberine	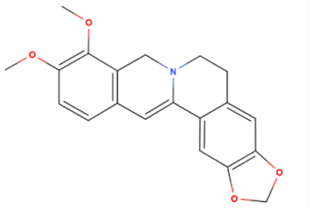	ND	22 ± 2	0.85 ± 0.45	0.012 ± 0.007
Berberrubine	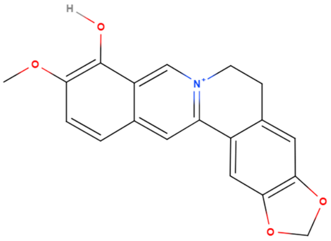	0.75 ± 0.10	43 ± 7	1.3 ± 0.4	0.085 ± 0.03
Thalifendine	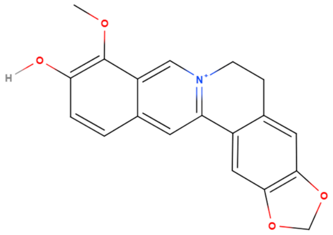	0.0083 ± 0.0008	ND	3.6 ± 1.4	0.73 ± 0.12
Columbamine		1360 ± 110	56 ± 19	1.4 ± 0.8	0.32 ± 0.13

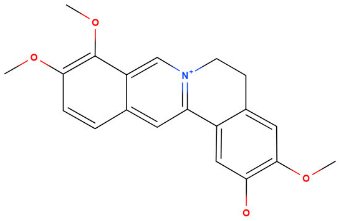

Jatrorrhizine	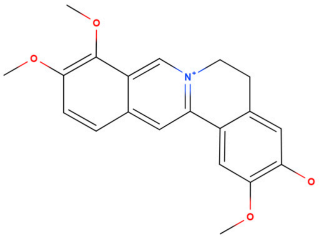	0.032 ± 0.014	0.004 ± 0.004	1.07 ± 0.25	0.59 ± 0.16
Demethyleneberberine	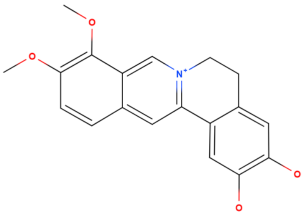	0.23 ± 0.04	0.98 ± 0.14	7.9 ± 2.7	0.12 ± 0.05
Dihydroxyberberine	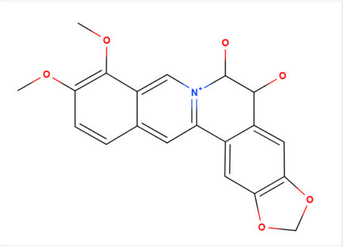	ND	ND	0.16 ± 0.06	0.0023 ± 0.0013
Dihydroxy-berberrubine (top) and Dihydroxy-thalifendine (bottom)	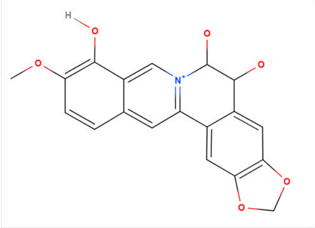 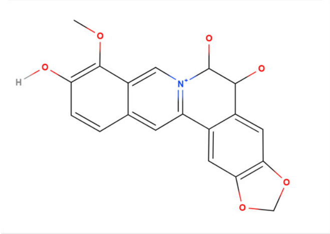	ND	0.14 ± 0.03#	0.25 ± 0.10#	0.25 ± 0.10#
Berberrubine glucuronide		ND	ND	ND	0.055 ± 0.013

	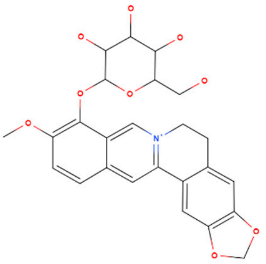				
Thalifendine glucuronide	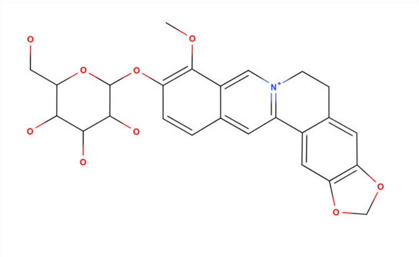	ND	ND	0.21 ± 0.12	0.06 ± 0.01
Jatrorrhizine glucuronide	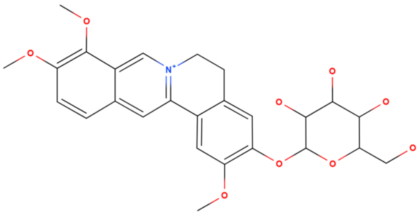	0.0023 ± 0.002	ND	0.050 ± 0.043	0.019 ± 0.007
Columbamine glucuronide	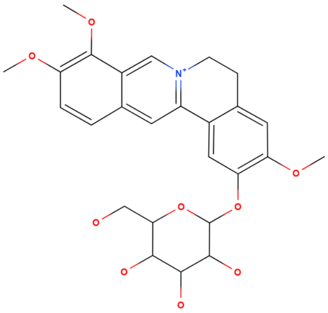	ND	ND	0.07 ± 0.04	0.007 ± 0.003
Demethyleneberberine glucuronide (DBG)	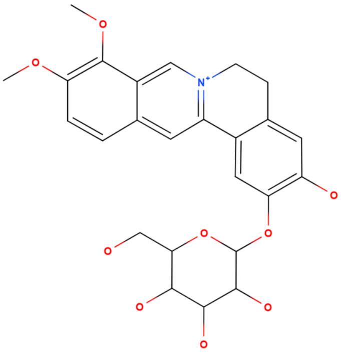	ND	ND	0.098 ± 0.044	0.0098 ± 0.0036
Demethyleneberberine sulfate (DB SO4)		0.27 ± 0.04	0.78 ± 0.10	2.4 ± 0.8	0.35 ± 0.05

	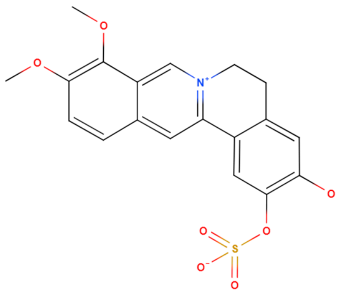				
Jatrorrhizine sulfate	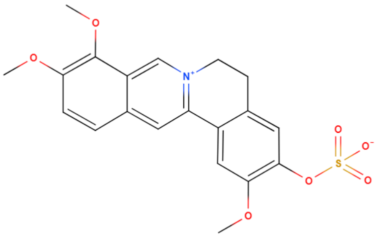	ND	0.098 ± 0.02	0.03 ± 0.01	0.0013 ± 0.0008
Berberrubine sulfate (top) and thalifendine sulfate (bottom) (Bbrb/Thalif SO4)	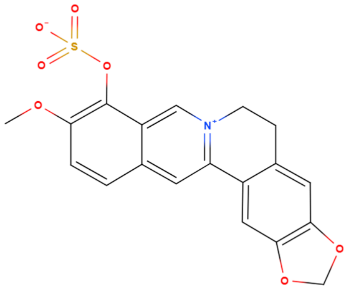 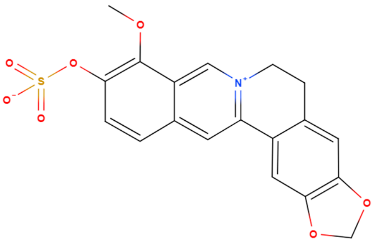	0.0035 ± 0.001#	0.046 ± 0.02#	1.4 ± 0.7#	0.17 ± 0.04#

ND = Not detected. Mean ± SEM reported. # Results reported are the sums of both compounds.

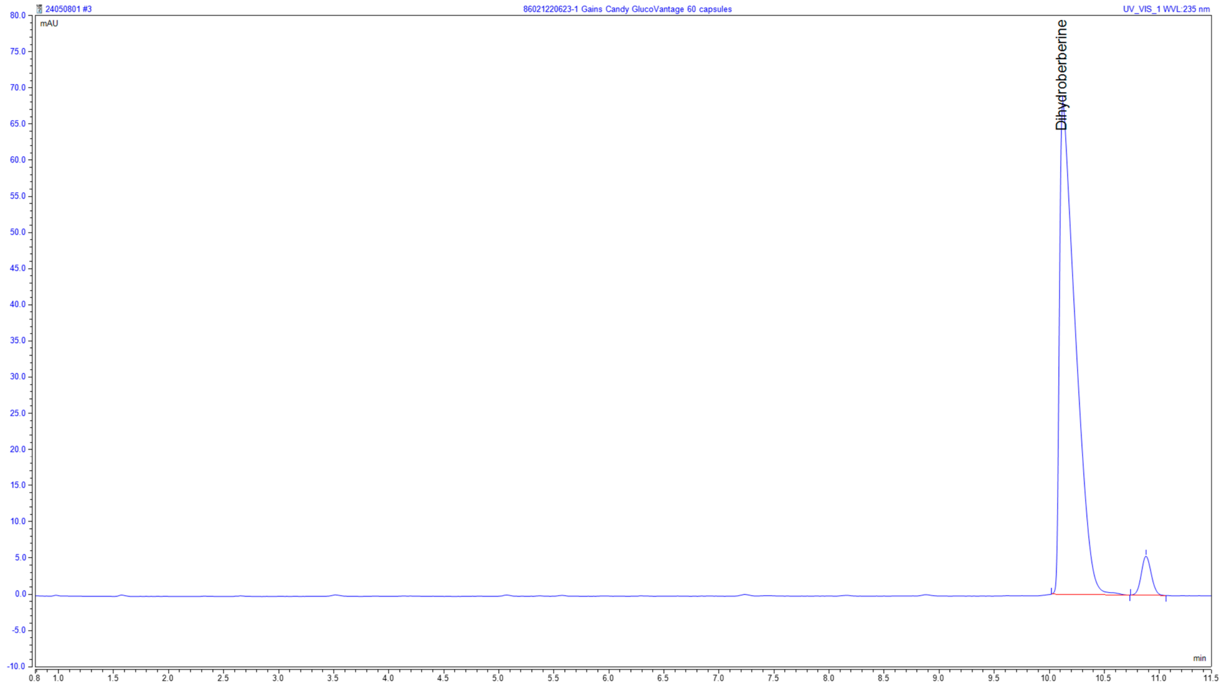
24050801 #3
86021220623-1 Gains Candy GlucoVantage 60 capsules
UV_VIS_1 WVL:235 nm
mAU
Dihydroberberine
min

(a)

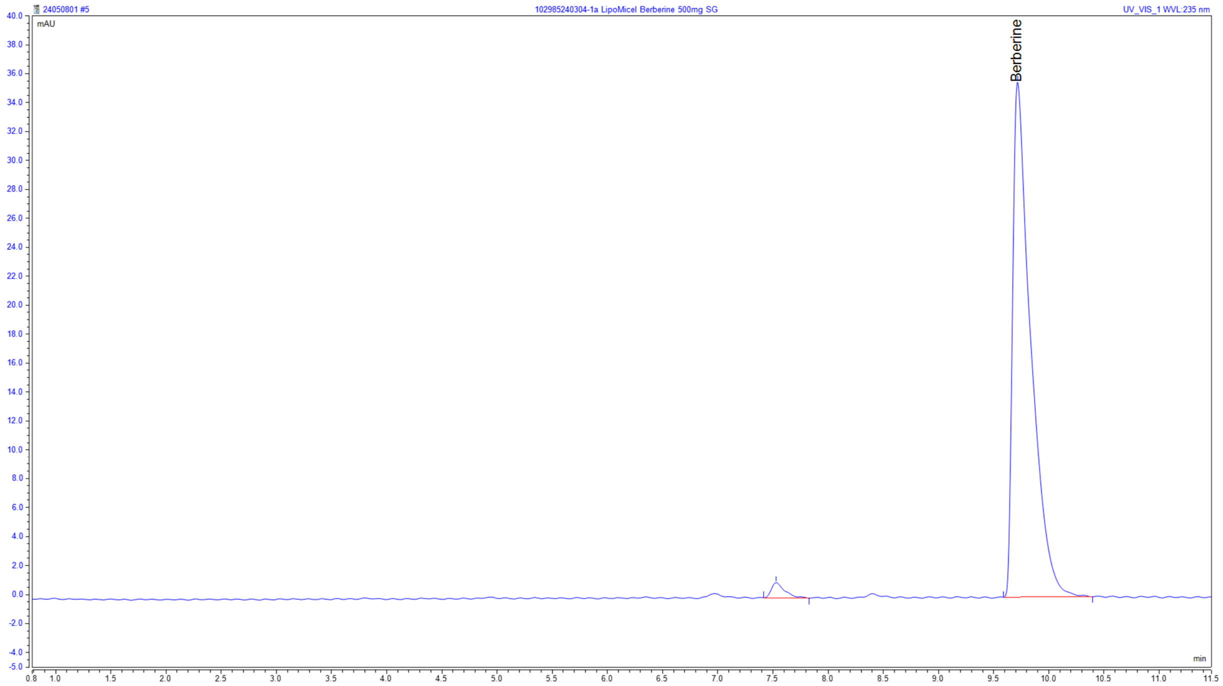
24050801 #5
102985240304-1a LipoMicel Berberine 500mg SG
UV_VIS_1 WVL:235 nm
mAU
Berberine
min

(b)

Figure S2: UHPLC chromatograms of study treatments. (a) DHB, chromatogram confirmed the identity and purity of dihydroberberine at its characteristic retention time of 10.12 minute with no detectable amount of berberine. The smaller peak at 10.9 minute likely resulted from the black pepper extract. (b) LMB, chromatogram confirmed the identity and purity of berberine at its characteristic retention time of 9.72 minute with no detectable amount of dihydroberberine. The smaller peak at 7.5 minute likely resulted from one of the excipients used to manufacture the capsule.
